# Antimicrobial Susceptibility of *Lactobacillus delbrueckii* subsp. *lactis* from Milk Products and Other Habitats

**DOI:** 10.3390/foods10123145

**Published:** 2021-12-18

**Authors:** Noam Shani, Simone Oberhaensli, Hélène Berthoud, Remo S. Schmidt, Hans-Peter Bachmann

**Affiliations:** 1 Competence Division Methods Development and Analytics, Agroscope, Schwarzenburgstrasse 161, 3003 Bern, Switzerland; helene.berthoud@agroscope.admin.ch; 2 Interfaculty Bioinformatics Unit and SIB Swiss Institute of Bioinformatics, University of Bern, Baltzerstrasse 6, 3012 Bern, Switzerland; simone.oberhaensli@bioinformatics.unibe.ch; 3 Research Division Food Microbial Systems, Agroscope, Schwarzenburgstrasse 161, 3003 Bern, Switzerland; remo.schmidt@agroscope.admin.ch (R.S.S.); hans-peter.bachmann@agroscope.admin.ch (H.-P.B.)

**Keywords:** antimicrobial susceptibility, *Lactobacillus delbrueckii*, broth microdilution, kanamycin, genome sequencing, SNP-based phylogeny

## Abstract

As components of many cheese starter cultures, strains of *Lactobacillus delbrueckii* subsp. *lactis* (*LDL*) must be tested for their antimicrobial susceptibility to avoid the potential horizontal transfer of antibiotic resistance (ABR) determinants in the human body or in the environment. To this end, a phenotypic test, as well as a screening for antibiotic resistance genes (ARGs) in genome sequences, is commonly performed. Historically, microbiological cutoffs (MCs), which are used to classify strains as either ‘sensitive’ or ‘resistant’ based on the minimal inhibitory concentrations (MICs) of a range of clinically-relevant antibiotics, have been defined for the whole group of the obligate homofermentative lactobacilli, which includes *LDL* among many other species. This often leads to inaccuracies in the appreciation of the ABR status of tested *LDL* strains and to false positive results. To define more accurate MCs for *LDL*, we analyzed the MIC profiles of strains originating from various habitats by using the broth microdilution method. These strains’ genomes were sequenced and used to complement our analysis involving a search for ARGs, as well as to assess the phylogenetic proximity between strains. Of *LDL* strains, 52.1% displayed MICs that were higher than the defined MCs for kanamycin, 9.9% for chloramphenicol, and 5.6% for tetracycline, but no ARG was conclusively detected. On the other hand, all strains displayed MICs below the defined MCs for ampicillin, gentamycin, erythromycin, and clindamycin. Considering our results, we propose the adaptation of the MCs for six of the tested clinically-relevant antibiotics to improve the accuracy of phenotypic antibiotic testing.

## 1. Introduction

The thermophilic lactic acid bacterium (LAB) *Lactobacillus delbrueckii* is important in many traditional fermented foods prepared worldwide (Table 1), and three subspecies (*bulgaricus*, *delbrueckii*, and *lactis*) have a long history of safe use [1]. To date, six subspecies have been described: *L. delbrueckii* subsp. *bulgaricus* (*LDB*), *L. delbrueckii* subsp. *delbrueckii* (*LDD*), *L. delbrueckii* subsp. *indicus* (*LDI*), *L. delbrueckii* subsp. *jakobsenii* (*LDJ*), *L. delbrueckii* subsp. *lactis* (*LDL*), and *L. delbrueckii* subsp. *sunkii* (*LDS*). In dairy fermentations, *LDB* is mainly used in yogurt making, whereas *LDL* is traditionally used in the production of cooked cheeses, owing its tolerance to the high temperatures during the early phases of cheese manufacturing. Both subspecies seem to have developed a similar adaptation towards optimized utilization of milk resources through reductive evolution and limited acquisition of particular functions. In its evolutionary path, *LDB* lost more functions than *LDL* and has thus further diverged from their common ancestor [2].

In yogurt fermentation, the so-called protocooperation between *Streptococcus salivarius* subsp. *thermophilus* and *LDB*, both of which grow in a mutualistic interaction by taking advantage of each other’s metabolism, has long been described [3]. Recent findings have indicated that in cheese, a similar mutualistic interaction may take place between *S. salivarius* subsp. *thermophilus* and *LDL* [4]. The production of semi-hard (e.g., Appenzeller^®^ and Tête de Moine PDO), hard (e.g., Emmentaler PDO, Le Gruyère PDO, and Comté PDO), and extra-hard cooked cheeses (e.g., Parmigiano Reggiano PDO, Grana Padano PDO, and Sbrinz PDO) relies on the acidification of milk by thermophilic starter cultures containing a combination of thermophilic LAB. A recent metagenomics study has revealed the presence of *S. salivarius* subsp. *thermophilus*, *Lactobacillus helveticus* and *LDL* in natural whey cultures used to produce Le Gruyère PDO [5]. Defined starter cultures are relatively commonly exclusively composed of *S. salivarius* subsp. *thermophilus* and *LDL*. The latter is responsible for the second phase of lactic acid fermentation given its lower pH optimum and its ability to ferment galactose besides glucose. *LDL* is thus of primary importance in the production of most types of cooked cheese [6]. Given its considerable peptidase complement and its cell envelope-associated proteinase activity, it contributes significantly to proteolysis. These characteristics, together with its propensity to undergo autolysis, confers *LDL* the ability to influence the development of flavor and texture during cheese ripening [7,8,9].

Similar to most LAB that constitute starter cultures, new strains of *LDL* and *LDB* used in food or feed applications are exempt of a full safety assessment, due to their qualified presumption of safety (QPS) status [10]. Nonetheless, several safety aspects must still be monitored, one of them being the absence of acquired resistance to clinically relevant antimicrobials [11]. The spread of antibiotic resistance genes (ARGs) has become a major issue in the last decades, threatening the efficacy of antibiotics used for medical purposes. Thus, limiting the dissemination of ARGs is an important measure, besides others, to prevent the development of antibiotic resistant bacteria [12]. It is in this respect that the bacteria introduced into the food chain through fermented foods become a concern. Indeed, fermented foods serve as a vehicle for bacteria to enter the body, where the exchange of genes, including ARGs, has been reported to occur [13,14,15]. To prevent the spread of ARGs through fermented foods, the European Food Safety Authority (EFSA) requires that strains deliberately introduced into the food chain should be devoid of acquired transferable antibiotic resistance (ABR) determinants [16]. Consequently, producers of bacterial cultures have to determine the ABR status of the strains they bring onto the market, even those belonging to species with a QPS status and meant for food production [10].

The EFSA Panel on Additives and Products or Substances used in Animal Feed (FEEDAP) recommends testing bacteria intended to be introduced into the food chain for nine antibiotics selected for their human and veterinary importance. To this end, minimal inhibitory concentrations (MICs) of these antibiotics should be determined using a standardized and internationally recognized phenotypic test [17]. For each antibiotic, a microbiological cutoff (MC) has been defined for several LAB taxa based on the available data. MCs are used as a basis to distinguish the strains with acquired antibiotic resistance from the susceptible ones. Although the absence of ARGs is already a good indication of a strain’s safety in terms of ABR, phenotypic tests allow for the detection of unknown ABR mechanisms or of common mechanisms that could not be detected with genomic assessment due, e.g., to potential methodological issues.

Because no MCs have been defined specifically for *L. delbrueckii* or its subspecies, MICs obtained for strains of *LDL* have to be compared with the MCs defined for obligate homofermentative lactobacilli, which include dozens of different phylogenetically related species [18]. The necessity of grouping all these species to define their MCs probably stems from the scarcity of ABR data for *L. delbrueckii* and other obligate homofermentative species; however, this approach could eventually lead to inaccuracies. For culture producers, this may have serious consequences, such as putting aside their assortment strains with interesting technological properties or introducing into the market strains with undetected ABR [19].

This study aimed to challenge the current MCs for *L. delbrueckii*, more specifically for *LDL*. To this end, we assessed antibiotic susceptibility at the genomic and phenotypic levels for 100 strains of *L. delbrueckii* isolated from various habitats. Based on the measured MICs and our calculations, we propose the revision of the recommended MCs for *LDL* for several antimicrobials.

**Table 1:** Subspecies of *Lactobacillus delbrueckii* isolated from various fermented food products (reviewed by [7,20,21]).

Product	Substrate	Subspecies of *Lactobacillus delbrueckii*	Country
Cheese	Animal milk	*L. delbrueckii* subsp. *delbrueckii, L. delbrueckii* subsp. *lactis*	Worldwide
Dahi	Cow/buffalo milk, starter culture	*L. delbrueckii* subsp. *indicus*	India, Nepal, Sri Lanka, Bangladesh, Pakistan
Misti dahi (mishti doi, lal dahi, payodhi)	Cow/buffalo milk	*L. delbrueckii* subsp. *bulgaricus*	India, Bangladesh
Tarag, Khoormog, Airag, Kumys	Cow/yak/goat/mare milk	*L. delbrueckii* subsp. *bulgaricus*	Mongolia
Yogurt	Animal milk	*L. delbrueckii* subsp. *bulgaricus*	Europe, Australia, America
Idli	Rice, black gram, or other dehusked pulses	*L. delbrueckii*	India, Sri Lanka, Malaysia, Singapore
Poto poto	Maize	*L. delbrueckii*	Congo
Sourdough	Rye, wheat	*L. delbrueckii*	Europe, Australia, America
Kimchi	Cabbage, green onion, hot pepper, ginger	*L. delbrueckii*	Korea
Kha Nhom Jeen	Rice	*L. delbrueckii*	Thailand
Soibum	Bamboo shoot	*L. delbrueckii*	India
Sunki	Turnip	*L. delbrueckii*	Japan
Tarhana	Wheat flour, yogurt, vegetables, spices	*L. delbrueckii*	Turkey
Tauco	Soybean	*L. delbrueckii*	Indonesia
Tsukemono	Pickled vegetable	*L. delbrueckii*	Japan

## 2. Materials and Methods

### 2.1. Bacterial Strains and Culture Conditions

A total of 101 strains of *L. delbrueckii* were selected for analysis (Table 2). Identification using matrix-assisted laser desorption–ionization time of flight (MALDI-TOF) and genome comparison based on the calculated average nucleotide identity (ANI) values (see *Identification using MALDI-TOF* and *Average Nucleotide Identities*) revealed inaccuracies in the taxonomic assignments. One strain assigned to another species was excluded from this study. After being re-assigned at the subspecies level, the remaining 100 *L. delbrueckii* strains (80 *LDL*, 17 *LDB*, and three *LDS*) were further analyzed. Seventy-eight strains obtained from various dairy products or dairy starter cultures, two from distilleries, one from human urine, one from human saliva, one from dried calf stomachs, one from fermented vegetables (sunki), and 16 others were of unknown origin. Six strains were constituents of commercial starter cultures used in the cheese industry.

The strains were stored at −80 °C in De Man, Rogosa, Sharpe (MRS) [22] broth with Tween 80 (Biolife Italiana Srl, Milan, Italy) containing sterile low-fat milk as the cryoprotectant. They were routinely cultured in MRS broth with Tween 80 at 37 °C under aerobic conditions. The absence of contaminants was confirmed by plating 10-fold serial dilutions in 10 mL 0.9% NaCl onto MRS agar plates and by observing the colony morphologies and the cells under a microscope.

### 2.2. Species-Level Identification

The identities of the strains were verified using MALDI-TOF on a MicroFlex™ LT/SH MS (Bruker Daltonics, Bremen, Germany), as described previously [23]. Data were acquired with FlexControl v. 3.4.105. Spectra were analyzed by the MBT Compass software v.1.4 (Bruker Daltonics, Inc., Billerica, MA, USA) and by Realtime Classification Biotyper MBT RUO 3.1 with BDAL v11.0 library.

### 2.3. Genome Sequences of the L. delbrueckii Strains

**Available assemblies were downloaded from the National Center for Biotechnology Information (NCBI).** Strains with unavailable assemblies in open databases were sequenced de novo. Their DNA was extracted as described previously [24]. Quality control assessment of the extracted DNA, library generation, and sequencing runs were performed on the Next Generation Sequencing Platform, University of Bern, Switzerland. In brief, the libraries were prepared using a TruSeq DNA PCR-free Library Prep kit (Illumina, 20015963, San Diego, CA, USA) in combination with TruSeq DNA UD Indexes (Illumina, 20022370) according to Illumina’s guidelines. Pooled DNA libraries were sequenced by paired-end sequencing (2 × 150 bp or 2 × 250 bp) either on an HiSeq 3000 instrument, or using a shared Illumina NovaSeq 6000 S Prime (SP) Reagent Kit (20029137; 500 cycles; Illumina, San Diego, CA, USA) on an Illumina NovaSeq 6000 instrument, generating an average of 4.3 million reads/library. ConFindr v.0.7.2 was used with raw reads to check the intra-species bacterial contamination [25]. Quality control of raw data was performed with FastQC v.0.11.7 [26]. Adaptor removal and trimming of raw data were performed using fastp v.0.20.0 [27]. The trimmed reads were assembled with SPAdes v.3.14.0 [28] in the --isolate mode, and contigs shorter than 200 bp were removed from the final assembly. QUAST v.4.6 [29] and BUSCO v.4.0.6 [30] were used in the --auto-lineage-prok mode to assess the quality of the assemblies. Newly obtained assemblies were uploaded to NCBI. Taxonomic affiliation of the assemblies was performed at the species level using the GTDB-Tk v.0.3.2 ‘classify’ workflow and reference data version r89 to confirm the previous identification. The Whole Genome Shotgun projects of the strains sequenced in this study have been deposited at GenBank under BioProject PRJNA777018 with GenBank WGS accessions JAJNSH000000000-JAJNVX000000000. The version described in this paper is version 01.

### 2.4. Average Nucleotide Identities (ANIs)

The assignment of the strains at the subspecies level was checked by calculating ANI values using fastANI [31]. Assemblies of the type strains of *LDD* (DSM 20074 ^T^ GenBank assembly accession GCA_001908495.1), *LDS* (JCM 17838 ^T^, GenBank assembly accession GCA_001888965.1), *LDI* (JCM 15610 ^T^, GenBank assembly accession GCA_001908415.1), and *LDJ* (DSM 26046 ^T^, GenBank assembly accession GCA_001888925.1) were included in the analysis. Visualization was done using the package gplots v.3.1.1 [32] using RStudio Pro v.1.4.1103-4 [33] and the R software v.4.0.3 [34].

### 2.5. Strains’ Phylogeny

Phylogenetic relationships of the isolates were assessed using PhaME v.1.0.2 [35] and IQ-TREE multicore v.2.0.3 [36], as described previously [19]. *LDL* and *LDB* were assessed separately. The resulting trees were visualized with the Interactive Tree Of Life (iTOL) v.5 [37]. Pairs of isolates with fewer than 50 single nucleotide polymorphisms (SNPs) in the SNP pairwise matrix from PhaME were considered to be possibly identical. In such cases, their origins and years of isolation were taken as an additional information to determine whether they should be considered different ‘strains in the taxonomic sense’ [38].

### 2.6. Screening Assemblies for ARGs

The genome assemblies were screened for known transferrable ABR genes with ABRicate [39] using the default filtering parameters (minimum DNA %identity = 75 and minimum DNA %coverage = 0) and with the databases NCBI AMRFinderPlus [40], CARD 2017 [41,42], ARG-ANNOT v.4 [43], and Resfinder v.3.0 [44]. The genome assembly of *P. acidilactici* FAM 13875 (GenBank assembly accession GCA_009789085.1), which harbors the ABR genes *tetM* and *ermA* [23], was included as positive control.

### 2.7. Antibiotic Susceptibility Testing and MC Determination

The existing MCs were evaluated based on the Standard Operating Procedure (SOP) 10.1 of the EUCAST [45]. The antibiotic susceptibility of the strains was tested at the Culture Collection of Switzerland (CCOS, Wädenswil, Switzerland) using a broth microdilution susceptibility method following the standard procedure of the International Organization for Standardization ISO 10932|IDF 223:2010 [46], as recommended by the FEEDAP [17]. In brief, the strains were propagated in MRS broth with Tween 80 (Biolife Italiana Srl, Milan, Italy) at 37 °C for 24 h and plated on MRS agar with Tween 80 (Biolife Italiana Srl, Milan, Italy). Standardized inocula were obtained by suspending colonies in a sterile saline solution to a turbidity of McFarland 1. The suspensions were then diluted 1000 times and transferred into microplates containing LAB susceptibility medium (LSM) [47] and different antibiotics of varying concentrations: ampicillin (AMP, 0.03–16 μg/mL), vancomycin (VAN, 0.25–128 μg/mL), gentamycin (GEN, 0.5–256 μg/mL), kanamycin (KAN, 2–1024 μg/mL), streptomycin (STR, 0.5–256 μg/mL), erythromycin (ERY, 0.016–8 μg/mL), clindamycin (CLI, 0.03–16 μg/mL), tetracycline (TET, 0.12–64 μg/mL), and chloramphenicol (CHL, 0.12–64 μg/mL). The microdilution plates were incubated at 37 °C for 48 h under anaerobic conditions. Growth was assessed visually.

The MIC distributions of the antibiotics were processed in the ECOFFinder program v2.1 (available at http://www.eucast.org/fileadmin/src/media/PDFs/EUCAST_files/MIC_distributions/ECOFFinder_XL_2010_v2.1_web_version.xlsm (accessed on 28 July 2021) [48] and the MCs were defined as the 99.0% ECOFF values. The MIC distributions and the MCs were visualized with the ggplot2 package v. 3.3.3 [49] using RStudio Pro v. 1.4.1103-4 [33] with the R software v. 4.0.3 [34].

## 3. Results

### 3.1. Taxonomic Assignment and ANIs

At the species level, the identity of most strains was confirmed using MALDI-TOF and GTDB-tk. Only strain CIRM BIA 879 was assigned to *L. johnsonii* and was excluded from the subsequent analyses.

Based on the ANI values, *LDB* formed a well-defined cluster, with pairs of strains displaying ANI similarities at 98.83–100% (median 99.19%), and it was evidently distinct from the other *L. delbrueckii* subspecies (Figure S1). By contrast, the *LDL* strains formed a quite inhomogeneous cluster (ANI 97.59–100%, median 98.79%), although it was still evidently divergent from the other *L. delbrueckii* subspecies.

The taxonomic assignment at the subspecies level was re-evaluated for three strains (Table 2): CIP 101810 and CIRM BIA 266 (both originally assigned to *LDL*) displayed a high similarity (98.27% and 98.61%, respectively) with the *LDS* type strain DSM 24966^T^, and CIRM BIA 1375 (originally *LDB*) displayed similarity with *LDL* DSM 20072^T^ (98.53%). NCIMB 702468 and NCIMB 702469, referenced in the NCIMB database as *LDS* and *LDD*, respectively, were assigned to *LDL*.

### 3.2. SNP-Based Phylogenetic Relationships of the Strains

**Figure 1:**
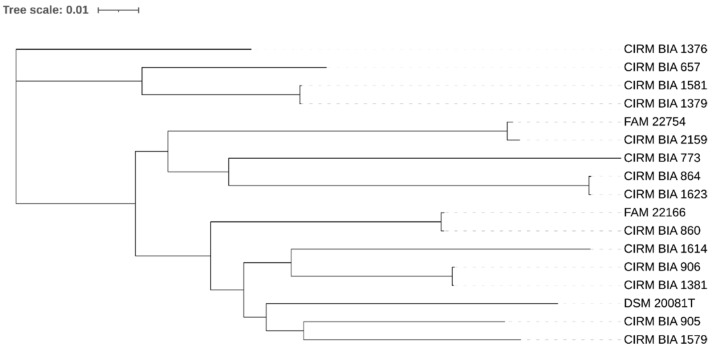
SNP-based phylogenetic tree of *Lactobacillus delbrueckii* subsp. *bulgaricus* strains assessed in this study. The scale shows the rate of single nucleotide polymorphisms.

The SNP-based analysis revealed that the *LDB* strains were all phylogenetically distinct (Figure 1). The closest pairs of strains displayed 34 (CIRM BIA 1379 and CIRM BIA 1581) and 94 (CIRM BIA 864 and CIRM BIA 1623) SNPs. As the origins of these isolates were different, they were considered closely related but different strains.

**Figure 2:**
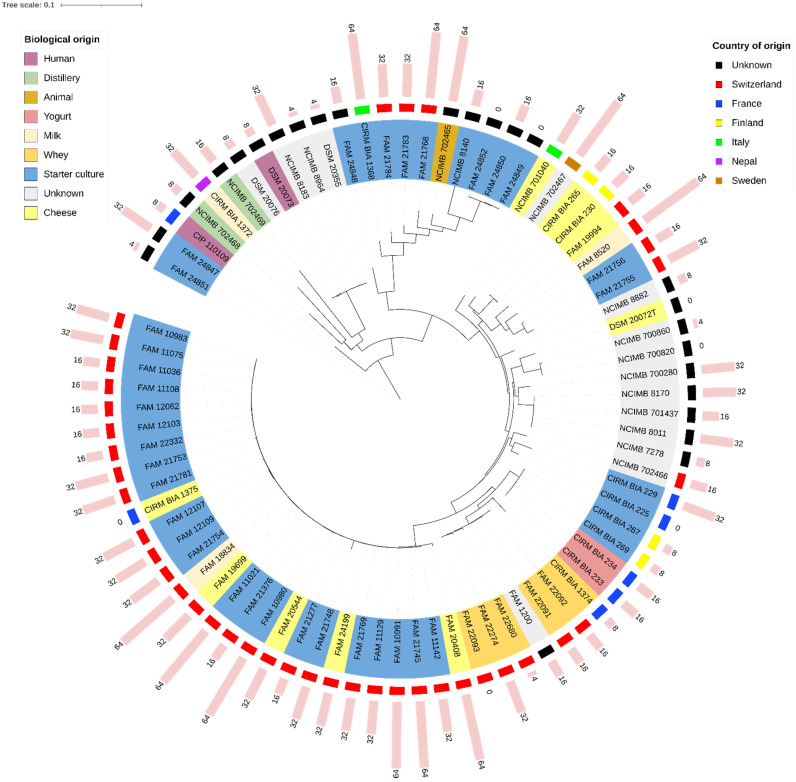
SNP-based phylogenetic tree of the *Lactobacillus delbrueckii* subsp. *lactis* strains assessed in this study. Country and biological origin, as well as the defined minimal inhibitory concentration of kanamycin (MICs, pink bars, along with their numerical values expressed in mg/L) are displayed. For strains that did not grow in the test medium or that have not been tested, MIC of kanamycin is indicated with ‘0′. The scale shows the rate of single nucleotide polymorphisms.

A more complex pattern was obtained for the *LDL* strains (Figure 2). Twenty-eight strains formed a homogeneous phylogenetic group. All pairs of strains in this group, however, displayed more than 120 SNPs (median 1067 SNPs). All but one (CIRM BIA 1375) were isolated from Swiss dairy products, mainly from cheese or from undefined mixed starter cultures, mostly obtained from different cheese factories. Several other groups consisted of similar, though different, strains: FAM 1200, FAM 22091, FAM 22092, FAM 22093, FAM 22274, and FAM 22680 displayed more than 368 SNPs. These strains were isolated from Swiss whey (no information available for FAM 1200). NCIMB 700820 and NCIMB 700860 displayed more than 2000 SNPs. Both CIRM BIA 230 and CIRM BIA 265 were isolated from Finnish Emmental cheese in 1968, but they were considered different strains, as they displayed 138 SNPs. CIRM BIA 233 and CIRM BIA 234 were both isolated from a French yogurt factory in 1984, and they displayed 12 SNPs. Given the strong suspicion that they belong to the same strain, CIRM BIA 234 was not included in the antibiotic susceptibility profiles. CIRM BIA 269 displayed 12 SNPs when compared with CIRM BIA 233 and CIRM BIA 234, but it was still regarded as a different strain given its different origin. CIRM BIA 267 had the same origin as CIRM BIA 269, but these isolates displayed more than 150 SNPs. NCIMB 8183, NCIMB 8964, DSM 20355, DSM 20073, and DSM 20076 all displayed more than 100 SNPs. NCIMB 7278 and NCIMB 701437 displayed 36 SNPs. As information about their origin was unavailable, they were considered identical, and only NCIMB 701437 was included in the antibiotic susceptibility profiles. NCIMB 8011 and NCIMB 8170, although considerably closely related to NCIMB 701437, were still considered different strains, as they displayed more than 50 SNPs with respect to the latter strain and between each other. Finally, FAM 24849, FAM 24850, and FAM 24852 all displayed fewer than 12 SNPs. As these strains were isolated from different commercial starter cultures but from the same company, there is a high probability that the same strain was used in all three starter cultures. Therefore, only FAM 24850 was included in the susceptibility profiles.

### 3.3. ARGs in the Genome Assemblies

The search for ARGs in the strain assemblies revealed a single match, namely strain FAM 22754. In the assembly of this strain, an *ermB* gene was detected in a small, low coverage, scaffold (763 bp). To confirm/infirm the presence of this gene in the strain FAM 22754, we performed a specific PCR targeting *ermB*, as described previously [50]. The presence of *ermB* could neither be confirmed using the DNA extracts used for sequencing, nor using new DNA extracts from the strain, and we concluded that its presence in the assembly was due to a contamination during DNA extraction, library preparation, or sequencing.

### 3.4. Antibiotic Susceptibility Profiles and Determination of MCs

Six strains (five *LDL* and one *LDB*) did not grow in the test medium (i.e., LSM): FAM 22093, CIRM BIA 225, NCIMB 700820, CIRM BIA 1375, DSM 20072 ^T^ and CIRM BIA 1381. Thus, they were excluded from subsequent analyses.

**Figure 3:**
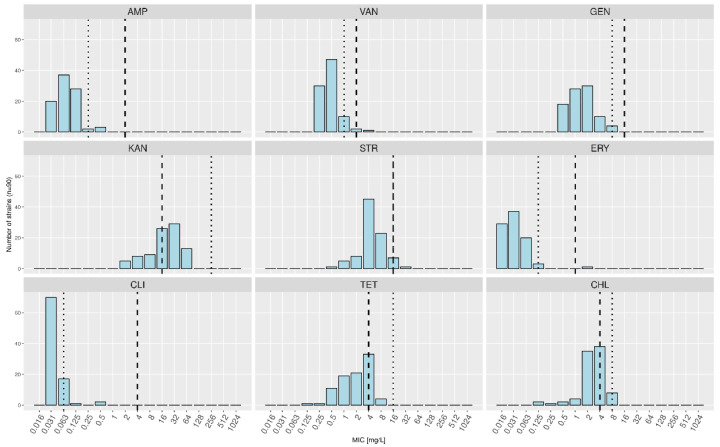
Combined minimal inhibitory concentration (MIC) distributions for *Lactobacillus delbrueckii* subsp. *lactis* and *L. delbrueckii* subsp. *bulgaricus*. The dashed lines indicate the current microbiological cutoffs (MCs) as defined by the European Food Safety Authority, and the dotted lines represent the calculated MCs based on the MICs measured in this study.

Among the 90 remaining *L. delbrueckii* strains, 42 (46.7%) displayed MICs higher than the FEEDAP MC for KAN, eight (8.9%) for CHL, four (4.4%) for TET, and one (1.1%) each for VAN, STR, and ERY (Table 3, Figure 3). The MCs for all the investigated subspecies were higher than the FEEDAP MCs for KAN (256 mg/L instead of 16 mg/L), TET (16 mg/L instead of 4 mg/L), and CHL (8 mg/L instead of 4 mg/L).

**Figure 4:**
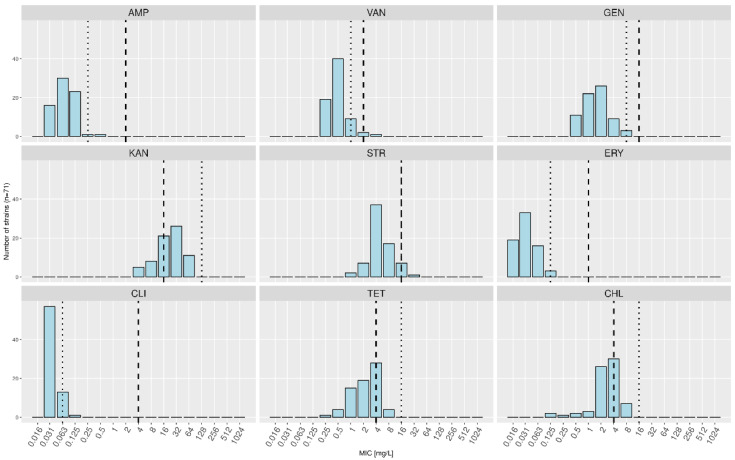
Minimal inhibitory concentration (MIC) distributions for *Lactobacillus delbrueckii* subsp. *lactis* only. The dashed lines indicate the current microbiological cutoffs (MCs) as defined by the European Food Safety Authority, and the dotted lines represent the calculated MCs based on the MICs measured in this study.

As for *LDL* (Table 3, Figure 4), 37 strains out of 71 (52.1%) displayed MICs higher than the FEEDAP MC for KAN, seven (9.9%) for CHL, four (5.6%) for TET, and one (1.4%) each for VAN and STR. The MCs calculated based solely on this subspecies were higher than the FEEDAP MCs for KAN (128 mg/L instead of 16 mg/L), TET (16 mg/L instead of 4 mg/L), and CHL (16 mg/L instead of 4 mg/L). Conversely, MCs were lower for AMP (0.25 mg/L instead of 2 mg/L), GEN (8 mg/L instead of 16 mg/L), ERY (0.125 mg/L instead of 1 mg/L), CLI (0.125 mg/L instead of 4 mg/L), and VAN (1 mg/L instead of 2 mg/L).

No link could be drawn between the levels of resistance to antibiotics and the origins of the strains or their position in the SNP-based phylogenetic tree (Figure 2).

## 4. Discussion

Used alongside *S. salivarius* subsp. *thermophilus*, *LDL* is the major *L. delbrueckii* subspecies added as a thermophilic starter in the production of cooked Swiss cheese varieties. The development of starter or adjunct cultures for the cheese market involves a thorough screening of strain characteristics. MIC values determined through broth microdilution testing are commonly used as an easy criterion for the early acceptance or rejection of candidate strains prior to the in-depth characterization of their properties to determine their suitability for cheese production. In the case of *LDL*, the ABR status of new candidates is often subject to debate, as values regularly exceed the defined FEEDAP MCs, particularly that for KAN (Agroscope, data not shown). The FEEDAP MCs have been defined for the entire group of obligate homofermentative lactobacilli, consisting of at least 31 species [18]. Here, we questioned the relevance of the current FEEDAP MCs for *L. delbrueckii*, more specifically for *LDL*.

An unambiguous identification of a strain is a prerequisite for any further safety assessment [17]. Controlling the taxonomic affiliation of our strain assortment led to the re-assignment of one and five strains at the species and subspecies levels, respectively. In addition, during the preliminary selection of strains suitable for this study, six strains in the Agroscope Culture Collection were taxonomically re-assigned at the subspecies level (data not shown). Here, we assessed the taxonomic affiliation at the subspecies level based on the ANI values. According to previous research, strains whose genomes display an ANI of ≥96.5% and an alignment fraction of ≥0.6 can be grouped into a single species [51]. At the subspecies level, however, no similar cutoffs have been defined. We therefore assessed visually the positions of the strains and their similarities in the ANI-based tree relative to different type strains. The subspecies of strains in some culture collections have often been defined based solely on physiological properties, and modern molecular techniques have not been systematically applied. Today, as NGS has become an affordable approach, taxonomic affiliations can be reassessed in the light of whole genome comparisons. Considering the taxonomic assessment performed here and the subsequent re-classifications, we strongly recommend that the taxonomic affiliation of the examined strains, even those deposited in culture collections, be systematically questioned. Comparisons at the genomic level are a good approach; however, more rapid methods that require less computational analysis (e.g., MALDI-TOF) may also provide a good evaluation of certain taxa. In our study, MALDI-TOF allowed for the discrimination of *LDL* and *LDB*, but not of *LDS*, as this subspecies is not yet referenced in the BDAL v11.0 library used here.

Most strains selected for analysis in our study were dairy isolates (obtained from milk, dairy products, natural whey, and other starter cultures) from Switzerland, as most strains were provided by the Agroscope Culture Collection, which includes strains mainly obtained from Swiss dairy products. To determine the MICs of the non-dairy isolates, we broadened the range of origins by including strains obtained from other habitats, such as human saliva, calf stomachs, or distilleries. Moreover, care was taken in expanding the strain selection to include those obtained from heterogeneous dairy products and those with different geographical origins. Furthermore, the selected strains were isolated within a period covering at least 69 years (1950–2019), within which the golden age of antibiotics and the subsequent dissemination of ABR determinants is included [52]. On the one hand, our strain selection allowed to avoid potential habitat-related biases. Although strains in some environments may be more exposed to selective pressure exerted by antibiotics or other antimicrobials, the spread of ABR is less likely to occur between habitats than within a single habitat [53,54]. Therefore, the overall elevated resistance towards KAN observed here is most likely an intrinsic characteristic of *L. delbrueckii* rather than a resistance that has been transmitted across habitats and time. On the other hand, selecting strains of different origins increases the chance of getting as many different strains as possible.

MCs were calculated using MIC distributions based on the susceptibility profiles of distinct strains. Here, potential duplicate strains were discarded after empirically examining the strains’ relationships in a SNP-based phylogenetic tree and after determining the number of SNPs between pairs of strains. This step is crucial to avoid duplicated MIC results that would lead to biased MIC distributions. The number of SNPs below which two strains were considered identical was defined subjectively based on previous SNPs analyses of different strains (data not shown) and may be adapted. Although the use of SNPs alone to discriminate between different strains is not standard, the threshold defined in this study is relatively consistent with results of other studies investigating clinical isolates [55,56,57]. Furthermore, the defined threshold of 50 SNPs appears reasonable considering the origins of the analyzed strains.

No association between the origins of the strains and their antimicrobial susceptibility patterns could be evidenced, suggesting that no particular antibiotic susceptibilities were selected in various ecological niches. Similarly, no link could be drawn between the year of isolation and the antimicrobial susceptibility patterns, suggesting that the selection pressure exerted by antibiotics used in veterinary medicine and farming towards *L. delbrueckii* has remained relatively moderate.

In this study, we postulated that the observed phenotypic resistances in *LDL* are actually false positives due to the inaccurate MCs. Our results tend to confirm our hypothesis. Indeed, although more than half of the tested *LDL* strains were phenotypically defined as ‘resistant’ to KAN according to the FEEDAP criteria, none of them displayed any known ARG. Similar observations were previously reported, with, e.g., 25% of tested *LDB* having MICs of KAN above the EFSA MC but no detected ARG [58]. The proportion of *L. delbrueckii* strains with MICs above the MCs for chloramphenicol (8.9%) in our study is higher than previously reported [58,59]. Conversely, the proportion of TET-resistant strains was lower in our study (4.4%) than in a previous study carried out on 11 *L. delbrueckii* strains [59]. The range of MICs of TET seems to be relatively broad by *L. delbrueckii*, as shown here, but also considering results from other studies. Indeed, the proportion of TET-resistant strains found here was lower (4.4%) than in a previous study carried out on 11 *L. delbrueckii* strains [59] but higher than in another one based on 4 *LDL* strains from natural whey starters, where no resistance to GEN, ERY, TET, and VAN was detected [60]. In addition to the fact that we detected no ARGs, all MIC distributions of *LDL* were unimodal, while a bimodal distribution would be expected in case of an acquired antibiotic resistance. On the other hand, the high susceptibilities to AMP, ERY, and CLI observed in most of our investigated strains represent a common feature of *Lactobacillus delbrueckii* that has already been reported by others [58,59,61]. For these three antibiotics, it rather seems that the MCs have been defined too high, which may open the door to false negative results and consequently the potential introduction of antibiotic resistant strains into the food chain.

Based on our observations, we therefore conclude that the MCs defined for the entire group of homofermentative lactobacilli should be redefined at the species or even at the subspecies level. Our strain selection may be too narrow in order that new MCs could be proposed for the entire species, as we have not tested some of the subspecies for their antimicrobial susceptibilities (*LDD*, *LDI,* and *LDJ*). However, our calculations based on strains of the subspecies *bulgaricus*, *lactis*, and *sunkii* suggest that the MCs for KAN, TET and CHL for *L. delbrueckii* should be increased, whereas the MCs for AMP, ERY and CLI should be decreased. For *LDL*, we suggest that the MCs for KAN, TET and CHL be increased to at least 128, 16, and 16 mg/L, respectively. Conversely, the MCs for AMP, ERY and CLI could be reduced to 0.25, 0.125, and 0.063 mg/L, respectively.

Recently, Stefanska et al. detected 32% of KAN-resistant strains in a wider range of LAB species using broth microdilution [62]. Of these strains, only three (4.6%) carried *aph(3′′)-IIIa*, a gene conferring resistance to this antibiotic. The issue raised in our study and that has already been reported for other species may thus be widespread in other LAB species, too [19,23].

The facts that a non-negligible number of strains were unable to grow in the test medium and that some variations occur in the phenotypic measures support the dual approach proposed by the FEEDAP, that is, phenotypic testing complemented by a search of the WGS for known ARGs [17]. The phenotypic approach may allow for the detection of yet unknown ABR mechanisms and is relatively advantageous in terms of cost and time. It can thus be used as a primary screening approach, along with the use of accurate MCs to avoid false positives that may drastically reduce the number of candidates [19]. Meanwhile, the WGS approach provides an appropriate method to evaluate strains that do not grow on test media and to confirm the resistances observed using the phenotypic test.

## Figures and Tables

**Table 2:** Bacterial strains and their taxonomic affiliation, origin, and year of isolation.

No.	Subspecies ^b^	Strain ID	Culture Collection ^a^	Origin	Year of Isolation	Original Depositor; Strain Designation	GenBank Assembly Accessions ^d^
1	*LDL*	FAM 1200	ACC	Unknown	Unknown	FAM 1200	**GCA_021135575.1**
2	*LDL*	FAM 8520	ACC	Swiss milk	Unknown	Jimeno, J.; FM 1	**GCA_021135595.1**
3	*LDL*	FAM 10980	ACC	Swiss undefined mixed starter culture	1979	Isolini, D.; Lb 101.07	**GCA_021135555.1**
4	*LDL*	FAM 10983	ACC	Swiss undefined mixed starter culture	1984	Isolini, D.; Lb 101.10	**GCA_021135485.1**
5	*LDL*	FAM 10991	ACC	Swiss undefined mixed starter culture	1980	Isolini, D.; Lb 104.80	**GCA_021135515.1**
6	*LDL*	FAM 11021	ACC	Swiss undefined mixed starter culture	1982	Isolini, D.; Lb 115.53	**GCA_021135535.1**
7	*LDL*	FAM 11036	ACC	Swiss undefined mixed starter culture	1979	Isolini, D.; Lb 119.17	**GCA_021135475.1**
8	*LDL*	FAM 11075	ACC	Swiss undefined mixed starter culture	1978	Isolini, D.; Lb 150.14	**GCA_021135455.1**
9	*LDL*	FAM 11108	ACC	Swiss undefined mixed starter culture	1983	Isolini, D.; Lb 157.02	**GCA_021135435.1**
10	*LDL*	FAM 11129	ACC	Swiss undefined mixed starter culture	1983	Isolini, D.; Lb 164.35	**GCA_021135405.1**
11	*LDL*	FAM 11142	ACC	Swiss undefined mixed starter culture	1988	Isolini, D.; Lb 202.02	**GCA_021135395.1**
12	*LDL*	FAM 12062	ACC	Swiss undefined mixed starter culture	1983	Isolini, D.; Lb 302.01	**GCA_021135375.1**
13	*LDL*	FAM 12103	ACC	Swiss undefined mixed starter culture	1986	Isolini, D.; Lb 325.13	**GCA_021135355.1**
14	*LDL*	FAM 12107	ACC	Swiss undefined mixed starter culture	1988	Isolini, D.; Lb 202.07	**GCA_021135335.1**
15	*LDL*	FAM 12109	ACC	Swiss undefined mixed starter culture	1988	Isolini, D.; Lb 202.09	**GCA_021135275.1**
16	*LDL*	FAM 18834	ACC	Swiss milk	2005	Unknown; C1	**GCA_021135295.1**
17	*LDL*	FAM 19699	ACC	Swiss Emmental cheese	1989–1990	Isolini, D.; 23.10	**GCA_021135315.1**
18	*LDL*	FAM 19994	ACC	Swiss Emmental cheese	1989–1990	Isolini, D.; 43.13	**GCA_021135255.1**
19	*LDL*	FAM 20408	ACC	Swiss hard cheese	1989–1990	Isolini, D.; LDELA 871-33	**GCA_021135235.1**
20	*LDL*	FAM 20544	ACC	Swiss hard cheese	1989–1990	Isolini, D.; LDEBU 927-84	**GCA_021135205.1**
21	*LDL*	FAM 21277	ACC	Swiss undefined mixed starter culture	Unknown	Meyer, J.; 101/100	GCA_005864055.1
22	*LDL*	FAM 21376	ACC	Swiss undefined mixed starter culture	Unknown	Meyer, J.; 169/126	**GCA_021135175.1**
23	*LDL*	FAM 21745	ACC	Swiss undefined mixed starter culture	1979	Isolini, D.; Lb 101.01	**GCA_021135195.1**
24	*LDL*	FAM 21748	ACC	Swiss undefined mixed starter culture	1979	Isolini, D.; Lb 101.56	**GCA_021135155.1**
25	*LDL*	FAM 21753	ACC	Swiss undefined mixed starter culture	1981	Isolini, D.; Lb 124.49	**GCA_021135095.1**
26	*LDL*	FAM 21754	ACC	Swiss undefined mixed starter culture	1982	Isolini, D.; Lb 153.48	**GCA_021135135.1**
27	*LDL*	FAM 21755	ACC	Swiss undefined mixed starter culture	1982	Isolini, D.; Lb 153.08	**GCA_021135105.1**
28	*LDL*	FAM 21756	ACC	Swiss undefined mixed starter culture	1982	Isolini, D.; Lb 153.09	**GCA_021135075.1**
29	*LDL*	FAM 21768	ACC	Swiss undefined mixed starter culture	1982	Isolini, D.; Lb 153.20	**GCA_021135055.1**
30	*LDL*	FAM 21769	ACC	Swiss undefined mixed starter culture	1978	Isolini, D.; Lb 150.10	**GCA_021135025.1**
31	*LDL*	FAM 21781	ACC	Swiss undefined mixed starter culture	1981	Isolini, D.; Lb 124.09	**GCA_021135015.1**
32	*LDL*	FAM 21783	ACC	Swiss undefined mixed starter culture	1982	Isolini, D.; Lb 153.27	**GCA_021134955.1**
33	*LDL*	FAM 21784	ACC	Swiss undefined mixed starter culture	1982	Isolini, D.; Lb 153.28	GCA_005864125.1
34	*LDL*	FAM 22091	ACC	Swiss natural whey culture	1973	A50.2	**GCA_021134995.1**
35	*LDL*	FAM 22092	ACC	Swiss natural whey culture	1977	A56.1	**GCA_021134975.1**
36	*LDL* ^c^	FAM 22093	ACC	Swiss natural whey culture	1977	A66.5	**GCA_021134935.1**
37	*LDL*	FAM 22274	ACC	Swiss natural whey culture	1968	A44.2	**GCA_021134915.1**
38	*LDL*	FAM 22332	ACC	Swiss undefined mixed starter culture	Unknown	Weishaupt, C.; 313.1	**GCA_021134885.1**
39	*LDL*	FAM 22680	ACC	Swiss natural whey culture	1967	A77.5	**GCA_021134855.1**
40	*LDL*	FAM 24199	ACC	Swiss Tomme cheese	2017	Shani, N.; 55/8	**GCA_021134875.1**
41	*LDS* (*LDL*)	CIP 101810	CIP	Unknown	Unknown	Unknown	**GCA_021134835.1**
42	*LDL*	CIP 110109	CIP	Human urine, France	1976	Vandekerkove; 103-76	**GCA_021134765.1**
43	*LDL* ^c^	CIRM BIA 225	CIRM	Artisanal lactic starter (for Gruyère de Comté cheese making), Franche-Comté, France	1963	Accolas, J.P.; P12	**GCA_021134795.1**
44	*LDL*	CIRM BIA 229	CIRM	Artisanal lactic starter (for Gruyère de Comté cheese making), Franche-Comté, France	1964	Accolas, J.P.; H5a	**GCA_021134815.1**
45	*LDL*	CIRM BIA 230	CIRM	Cheese (Emmental), Finland	1968	CNRZ331	**GCA_021134745.1**
46	*LDL*	CIRM BIA 233	CIRM	Probably a French yoghurt factory	1984	Cluzel, P.J.; LT4-G2N	**GCA_021134735.1**
47	*LDL*	CIRM BIA 234	CIRM	Probably a French yoghurt factory	1984	Cluzel, P.J.; LT4-G18	**GCA_021134695.1**
48	*LDL*	CIRM BIA 265	CIRM	Cheese (Emmental), Finland	1968	CNRZ330	**GCA_021134675.1**
49	*LDS* (*LDL*)	CIRM BIA 266	CIRM	Fermented milk (kefir), Russia	1971	Accolas, J.P.; KFA1	**GCA_021134645.1**
50	*LDL*	CIRM BIA 267	CIRM	Lactic starter (for Emmental cheese making), Finland	1974	Tybeck, E., LKT; VALIO	**GCA_021134615.1**
51	*LDL*	CIRM BIA 269	CIRM	Artisanal lactic starter (for Emmental cheese making), Finland	Unknown	Tybeck, E.; ISL 19	**GCA_021134635.1**
52	*LDL*	CIRM BIA 1368	CIRM	Starter (for Grana Padano cheese making), Piedmont, Italy	1988	IMPC Al	**GCA_021134595.1**
53	*LDL*	CIRM BIA 1372	CIRM	Yak milk, Nepal	1996	Quenee, P.; Np 5t	**GCA_021134505.1**
54	*LDL*	CIRM BIA 1374	CIRM	Whey (from Comté), Franche-Comté, France	1993	CML10	**GCA_021134545.1**
55	*LDL* ^c^	DSM 20072 ^T^	DSMZ	Emmental cheese (country of origin unknown)	Before 22.08.1990	Snog-Kjaer, A. (Orla-Jensen, S., *Thermobacterium lactis* No. 10)	GCA_002278095.1
56	*LDL*	DSM 20073	DSMZ	Saliva (country of origin unknown)	Before 22.08.1990	Williams, N.; 14-1	**GCA_021134535.1**
57	*LDL*	DSM 20076	DSMZ	Unknown	Before 22.08.1990	Fred, E.B.; F 59	**GCA_021134495.1**
58	*LDL*	DSM 20355	DSMZ	Unknown	Before 22.08.1990	McCoy; Ld 5	**GCA_021134475.1**
59	*LDL*	NCIMB 7278	NCIMB	Unknown	Before 01.01.1950	Dorner, W.; 39 E/K	**GCA_021134455.1**
60	*LDL*	NCIMB 8011	NCIMB	Unknown	Before 07.02.1950	Dorner	**GCA_021134375.1**
61	*LDL*	NCIMB 8140	NCIMB	“Ga” starter culture	Before 01.09.1956	“““Ga”””	**GCA_021134435.1**
62	*LDL*	NCIMB 8170	NCIMB	Unknown	Before 1999	Merck & Co., Inc.; MB 367	**GCA_021134415.1**
63	*LDL*	NCIMB 8183	NCIMB	Unknown	Before 30.11.1950	McCoy, E.; 326	**GCA_021134395.1**
64	*LDL*	NCIMB 8882	NCIMB	Unknown	Before 01.07.1957	Winkler, K.C.; 1175	**GCA_021134335.1**
65	*LDL*	NCIMB 8964	NCIMB	Unknown	Before 01.12.1958	Galloway & Barton-Wright	**GCA_021134315.1**
66	*LDL*	NCIMB 700280	NCIMB	Unknown	Before 01.01.1954	244	**GCA_021134355.1**
67	*LDL* ^c^	NCIMB 700820	NCIMB	Unknown	Before 01.01.1954	C808/5	**GCA_021134295.1**
68	*LDL*	NCIMB 700860	NCIMB	Unknown	Before 01.01.1956	18/40	**GCA_021134275.1**
69	*LDL*	NCIMB 701040	NCIMB	Italian hard cheese	Before 01.01.1957	C14/8	**GCA_021134235.1**
70	*LDL*	NCIMB 701437	NCIMB	Unknown	Unknown	Snog-Kjaer, A.; LI 1	**GCA_021134215.1**
71	*LDL*	NCIMB 702465	NCIMB	Dried calve stomachs for Gruyere cheese	Unknown	L24, C57	**GCA_021134255.1**
72	*LDL*	NCIMB 702466	NCIMB	Switzerland	Unknown	Ritter, P.; L26, H14, 1304	**GCA_021134175.1**
73	*LDL*	NCIMB 702467	NCIMB	Sweden	Before 01.01.1981	Swartling, P.; L27, L39 (WL39)	**GCA_021134185.1**
74	*LDL* (*LDS*)	NCIMB 702468	NCIMB	Distillery	Before 01.01.1961	Sharpe, M.E.; M2/2	**GCA_021134155.1**
75	*LDL* (*LDD*)	NCIMB 702469	NCIMB	Distillery	Before 01.01.1961	Sharpe, M.E.; LE8, M2/3	**GCA_021134135.1**
76	*LDL*	FAM 24847	NC	Commercial cheese starter culture	2019	na	**GCA_021134095.1**
77	*LDL*	FAM 24848	NC	Commercial cheese starter culture	2019	na	**GCA_021134115.1**
78	*LDL*	FAM 24849	NC	Commercial cheese starter culture	2019	na	**GCA_021134075.1**
79	*LDL*	FAM 24850	NC	Commercial cheese starter culture	2019	na	**GCA_021134035.1**
80	*LDL*	FAM 24851	NC	Commercial cheese starter culture	2019	na	**GCA_021134055.1**
81	*LDL*	FAM 24852	NC	Commercial cheese starter culture	2019	na	**GCA_021134005.1**
82	*LDB*	FAM 22166	ACC	Swiss natural whey culture	1968	A135.3	**GCA_021133995.1**
83	*LDB*	FAM 22754	ACC	Yoghurt	1952	A171.1	**GCA_021133975.1**
84	*LDB*	CIRM BIA 657	CIRM	Fermented milk, Crete, Greece	1987	Zourari, A.; ZL071B1	**GCA_021133935.1**
85	*LDB*	CIRM BIA 773	CIRM	Lactic starter (for yoghurt making), Île-de-France, France	1963	Chevalier, R.; LT1	**GCA_021133955.1**
86	*LDB*	CIRM BIA 860	CIRM	Lactic starter (for yoghurt making), Rhône-Alpes, France	1971	Accolas, J.P.; LAY 1	**GCA_021133915.1**
87	*LDB*	CIRM BIA 864	CIRM	Fermented milk, France	Unknown	IL1609	**GCA_021133885.1**
88	*Lactobacillus johnsonii* (*LDB*)	CIRM BIA 879	CIRM	Tarag (yoghurt), Mongolia	1974	Accolas, J.P.	na
89	*LDB*	CIRM BIA 905	CIRM	Cheese (Boulettes d’Avesne), Nord-Pas-de-Calais, France	1998	Quenee, P.; AV2	**GCA_021133875.1**
90	*LDB*	CIRM BIA 906	CIRM	Yak milk, Nepal	1996	Quenee, P.; NP 2T	**GCA_021133835.1**
91	*LDL* (*LDB*) ^c^	CIRM BIA 1375	CIRM	Cheese (Morbier, raw milk), Jura, France	1993	10F10	**GCA_021133855.1**
92	*LDB*	CIRM BIA 1376	CIRM	Lactic starter (for yoghurt making), Bulgaria	1978	Bouillanne, C. 5	**GCA_021133815.1**
93	*LDB*	CIRM BIA 1379	CIRM	Yoghurt (ewe milk), Crete, Greece	1987	Zourari, A.; ZL023A1	**GCA_021133775.1**
94	*LDB* ^c^	CIRM BIA 1381	CIRM	Yoghurt, Bali, Indonesia	1990	CNRZ1493	**GCA_021133785.1**
95	*LDB*	CIRM BIA 1579	CIRM	Lactic starter, USA	1974	Reinbold, G.W.; LB C	**GCA_021133715.1**
96	*LDB*	CIRM BIA 1581	CIRM	Artisanal yoghurt, Crete, Greece	1987	Zourari, A.K; ZL031B4	**GCA_021133685.1**
97	*LDB*	CIRM BIA 1614	CIRM	Yoghurt, Île-de-France, France	Unknown	Chevalier, R.; LY3	**GCA_021133675.1**
98	*LDB*	CIRM BIA 1623	CIRM	Yoghurt, the Netherlands	Unknown	NIZO Ib	**GCA_021133735.1**
99	*LDB*	CIRM BIA 2159	CIRM	Yoghurt (Bulgarian), Belgium	1960	Accolas, J.P.; RM7	**GCA_021133755.1**
100	*LDB*	DSM 20081 ^T^	DSMZ	Bulgarian yoghourt (country of origin unknown)	Before 22.08.1990	Orla-Jensen, S.; 14	GCA_000056065.1
101	*LDS*	DSM 24966 ^T^	DSMZ	Sunki, Japan	2004	K. Watanabe; YIT 11221	GCA_001888965.1

^a^ ACC: Agroscope Culture Collection, Agroscope, Bern, Switzerland; CIRM: Centre International de Ressources Microbiennes, Bactéries d’Intérêt Alimentaire, Institut National de la Recherche Scientifique (INRA), Rennes, France; DSMZ: German Collection of Microorganisms and Cell Cultures GmbH, Braunschweig, Gemany; NC: not communicated (private company); NCIMB: National Collection of Industrial Food and Marine Bacteria, Aberdeen, Scotland, UK. ^b^ Taxon as determined in this study. *LDL*: *Lactobacillus delbrueckii* subsp. *lactis*; *LDB*: *L. delbrueckii* subsp. *bulgaricus*; *LDD*: *L. delbrueckii* subsp. *delbrueckii*; *LDS*: *L. delbrueckii* subsp. *sunkii*. If the assignment of the strains in the present study diverged from the official denomination, the taxon as referenced in the collections is indicated in brackets. ^c^ No growth in test medium LSM. ^d^ Bolded accession numbers indicate the strains that have been sequenced in this study (description in the Section 2). ^T^ Type strain. na: not available.

**Table 3:** Distributions of the minimal inhibitory concentrations of the analyzed strains. The bolded numbers indicate the number of strains for all subspecies analyzed (*Lactobacillus delbrueckii* subsp. *lactis*, *bulgaricus* and *sunkii*). The numbers of strains for each subspecies are indicated in brackets in this order: *lactis*, *bulgaricus*, *sunkii*. The microbiological cutoffs proposed by the European Food Safety Authority are indicated in brackets beside each antibiotic and expressed in mg/L. AMP: ampicillin; VAN: vancomycin; GEN: gentamycin; KAN: kanamycin; STR: streptomycin; ERY: erythromycin; CLI: clindamycin; TET: tetracycline; CHL: chloramphenicol.

	Minimal Inhibitory Concentrations (mg/L)																
Antibiotic	0.016	0.03	0.06	0.12	0.25	0.5	1	2	4	8	16	32	64	128	256	512	1024
AMP (2)		**20** (16/4/0)	**37** (30/6/1)	**28** (23/3/2)	**2** (1/1/0)	**3** (1/2/0)	0	0	0	0	0						
VAN (2)					**30** (19/10/1)	**47** (40/5/2)	**10** (9/1/0)	**2** (2/0/0)	**1** (1/0/0)	0	0	0	0	0			
GEN (16)						**18** (11/7/0)	28 (22/3/3)	**30** (26/4/0)	**10** (9/1/0)	**4** (3/1/0)	0	0	0	0	0		
KAN (16)								**5** (0/5/0)	**8** (5/3/0)	**9** (8/1/0)	**26** (21/3/2)	**29** (26/2/1)	**13** (11/2/0)	0	0	0	0
STR (16)						**1** (0/1/0)	**5** (2/3/0)	**8** (7/1/0)	**45** (37/5/3)	**23** (17/6/0)	**7** (7/0/0)	**1** (1/0/0)	0	0	0		
ERY (1)	**29** (19/9/1)	**37** (33/4/0)	**20** (16/2/2)	**3** (3/0/0)	0	0	0	**1** (0/1/0)	0	0							
CLI (4)		**70** (57/11/2)	**17** (13/4/0)	**1** (1/0/0)	0	**2** (0/1/1)	0	0	0	0	0						
TET (4)				**1** (0/1/0)	**1** (1/0/0)	**11** (4/7/0)	**19** (15/4/0)	**21** (19/1/1)	**33** (28/3/2)	**4** (4/0/0)	0	0	0				
CHL (4)				**2** (2/0/0)	**1** (1/0/0)	**2** (2/0/0)	**4** (3/1/0)	**35** (26/8/1)	**38** (30/6/2)	**8** (7/1/0)	0	0	0				

## Data Availability

Publicly available datasets were analyzed in this study. Genomic data generated in this study have been deposited at GenBank under BioProject PRJNA777018 with GenBank WGS accessions JAJNSH000000000-JAJNVX000000000. Other publicly available genomic data analysed in this study can be found at GenBank under following Genbank WGS accessions: GCA_005864055.1, GCA_005864125.1, GCA_002278095.1, GCA_000056065.1, GCA_001888965.1, GCA_001908495.1, GCA_001888965.1, GCA_001908415.1, GCA_001888925.1, and GCA_009789085.1.

## Supplementary Materials

The following are available online at https://www.mdpi.com/article/10.3390/foods10123145/s1, Figure S1: Average Nucleotide Identities of the *Lactobacillus delbrueckii* strains.
