# Supplementary figures and images for: Antimicrobial Susceptibility of Lactobacillus delbrueckii subsp. lactis from Milk Products and Other Habitats
Source: Foods. 2021 Dec 18;10(12):3145. doi: 10.3390/foods10123145 (PMC8701367; doi:10.3390/foods10123145)

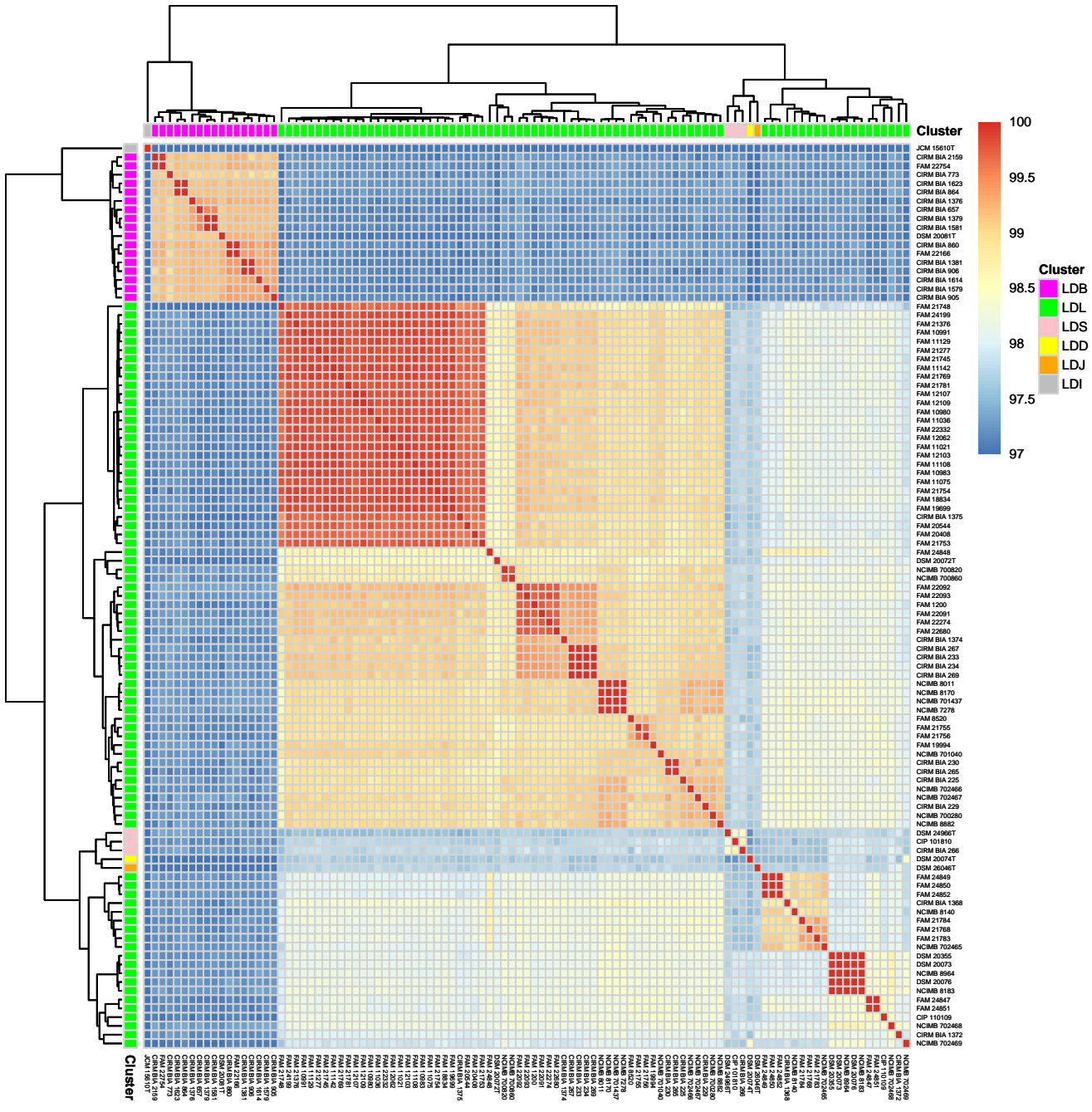

Supplement: Supplementary file 1 [file foods-10-03145-s001.zip › Fig_S1_LD_pub_pheatmap.pdf]
